# Mechanical and Fire Properties of Multicomponent Flame Retardant EPDM Rubbers Using Aluminum Trihydroxide, Ammonium Polyphosphate, and Polyaniline

**DOI:** 10.3390/ma12121932

**Published:** 2019-06-15

**Authors:** Benjamin Zirnstein, Dietmar Schulze, Bernhard Schartel

**Affiliations:** Bundesanstalt für Materialforschung und -prüfung (BAM), Unter den Eichen 87, 12205 Berlin, Germany; benjamin.zirnstein@bam.de (B.Z.); dietmar.schulze@bam.de (D.S.)

**Keywords:** EPDM, rubber, flame retardant, polyaniline, aluminum trihydroxide (ATH)

## Abstract

In this study, multicomponent flame retardant systems, consisting of ammonium polyphosphate (APP), aluminum trihydroxide (ATH), and polyaniline (PANI), were used in ethylene propylene diene monomer (EPDM) rubber. The multicomponent system was designed to improve flame retardancy and the mechanical properties of the rubber compounds, while simultaneously reducing the amount of filler. PANI was applied at low loadings (7 phr) and combined with the phosphorous APP (21 phr) and the mineral flame retardant ATH (50 phr). A comprehensive study of six EPDM rubbers was carried out by systematically varying the fillers to explain the impact of multicomponent flame retardant systems on mechanical properties. The six EPDM materials were investigated via the UL 94, limiting oxygen index (LOI), FMVSS 302, glow wire tests, and the cone calorimeter, showing that multicomponent flame retardant systems led to improved fire performance. In cone calorimeter tests the EPDM/APP/ATH/PANI composite reduced the maximum average rate of heat emission (MARHE) to 142 kW·m^−2^, a value 50% lower than that for the unfilled EPDM rubber. Furthermore, the amount of phosphorus in the residues was quantified and the mode of action of the phosphorous flame retardant APP was explained. The data from the cone calorimeter were used to determine the protective layer effect of the multicomponent flame retardant systems in the EPDM compounds.

## 1. Introduction

Ethylene propylene diene monomer rubber (EPDM) is a terpolymer, consisting of a saturated backbone with unsaturated side chains. Due to its unique properties, EPDM is the most widely used synthetic rubber for non-tire applications. It is used in many fields including construction, electrical insulation, and the automotive industry [1,2]. EPDM rubbers offer usability at low temperatures, excellent weather and chemical resistance, as well as a low dielectric constant [3]. Unlike saturated ethylene propylene monomer (EPM) rubber, EPDM rubbers are vulcanized with peroxides, forming C–C bonds or sulfur crosslink agents. Vulcanization with sulfur produces superior dynamical and mechanical properties.

A drawback of carbon-based rubber is that it burns easily, causing rapid fire spread and the emission of high amounts of dense smoke. To overcome this fire risk, high amounts of flame retardants are applied. Due to environmental and health issues in the past, halogen-free flame retardants are required. Mineral flame retardants and phosphorous flame retardants are the alternatives of choice [4,5,6]. Mineral flame retardants, such as aluminum trihydroxide (ATH) and magnesium hydroxide (MH), are used widely because of their low cost [7]. ATH acts through fuel replacement in the condensed phase and through fuel dilution and cooling in the gas phase, due to the release of water into the flame and the formation of a ceramic protective layer [8,9,10,11]. To achieve a sufficient flame retardancy effect, mineral fillers like ATH have to be used at high loadings, usually of 40–70%. These high loadings often deteriorate the physical and mechanical properties of the rubber compounds [12].

Phosphorous flame retardants have multiple modes of action and show gas phase and condensed phase activity. Phosphorous flame retardants increase the stability of the char by incorporating P atoms, often resulting in higher amounts of aromatic char with a glassy residue surface [5,13]. Furthermore, phosphorous flame retardants work in the gas phase by releasing molecules that contain phosphorus. These phosphorous molecules form radicals, which act as radical scavengers and cause flame inhibition [14,15]. Phosphorous flame retardants are used on the industrial scale, but, like mineral flame retardants, high loads are required, causing deterioration of the mechanical properties of the rubber.

Consequently, reducing the filler loading while boosting fire performance is the major target for flame retarded rubbers. Using multicomponent flame retardant systems, i.e., a combination of phosphorous flame retardants, mineral fillers, and/or nanocomposites, is a promising strategy to achieve this goal [8,16,17]. The synergistic effects on flame retardancy of phosphorous flame retardants and metal hydrates have been studied, revealing such effects as increased limiting oxygen index (LOI) and reduced heat emission in various materials, such as polyolefins [18,19,20,21] and rubber composites [22,23]. Combinations of ATH and ammonium polyphosphate (APP) are concentrated on producing the thermally stable P–Al–O structure surface coating. The combination of the film-forming action of ultraphosphate with the thermal stability of Al_2_O_3_ resulted in improved surface protection of the polymer [24].

To improve the performance of the flame retardants and reduce the filler loading, additional synergistic effects of the flame retardants were exploited. One synergism frequently discussed in flame retardancy is known as phosphorus–nitrogen synergism. P–N species are formed, which improve the protective layer effect due to increased thermal stability [25,26,27,28,29]. This causes reduced gas transport into the flame and decreased thermal feedback from the flame to the virgin material. Therefore, a reasonable approach to achieve a synergistic effect is a combination of a flame retardant containing phosphorus with a polymer containing nitrogen. A widely used nitrogenous polymer is polyaniline (PANI). The conducting polymer PANI is usually used in EPDM materials designed to absorb microwaves and radar [30,31,32]. In polystyrene and epoxy resins PANI revealed flame retardant properties in combination with montmorillonite [33,34,35,36]. In paper composites and epoxy resin, modified PANI or PANI in combination with additional fillers has been successfully applied as a flame retardant [37,38,39,40]. In EPDM rubber compounds PANI showed improved flame retardancy in combination with phosphorous flame retardants [41]. The overall fire performance of a multicomponent system depends strongly on the interaction of the flame retardant with the polymeric matrix [42,43,44].

In contrast to the former study, which primarily focused on the impact of PANI, this study investigates the performance of multicomponent flame retardant systems [41]. In this work the multicomponent flame retardant system consisting of the phosphorous flame retardant APP, the mineral filler ATH, and the polymeric synergist PANI was applied to EPDM. The study investigates the influence of the polyaromatic nitrogenous PANI as an effective flame retardant and potential char-forming agent in the multicomponent flame retardant system. The fillers were varied systematically. Six EPDM compounds were compounded and their physical and mechanical properties were analyzed. Furthermore, the impact of the fillers on ignitability and fire performance was evaluated. In addition, the protective layer effect of the multicomponent system on the EPDM compounds was quantified.

## 2. Materials and Methods

### 2.1. Materials

EPDM (Keltan® 2450, 48 wt.% and 4.1 wt.% of ethylene and ethylidene-norbornene, respectively), 2,2,4-trimethyl-1,2-dihydrochinolin (HS, Vulkanox HS/LG), zinc oxide (Zincoxyd aktiv®), thiuram MS (Rhenocure Thiuram MS/C), and cyclohexyl-2-benzothiazolsulfenamid (CZ, Vulkacit CZ/C) were supplied by Lanxess. Carbon black (CB, N550) was supplied by Evonik Industries AG, Essen, Germany. Sulfur was supplied by Merck, Darmstadt, Germany. Stearic acid (stearic acid pure) was obtained from Baerlocher, Unterschleißheim, Germany. Zinc oxide and stearic acid are activators, CZ and Thiuram MS are accelerators, and HS is an antioxidant. Polyaniline (PANI) was purchased from Chemos GmbH & Co. KG. (Altdorf, Germany) The flame retardant APP (Exolit® AP 766 (TP)) was supplied by Clariant (Muttenz, Switzerland), and ATH (aluminum trihydroxide, Apyral 200SM) was supplied by Nabaltec AG (Schwandorf, Germany).

### 2.2. Preparation of the EPDM Compounds

EPDM and other ingredients were mixed in an internal mixer (HF Mixing Group, Freudenberg, Germany) and afterward compounded on a two-roll mill (HF Mixing Group, Freudenberg, Germany). The ingredients of all rubber compounds are listed in Table 1. The rolls were set to a temperature of 50 °C, a speed of 8 rpm, and a friction of 1.1:1. The compounds were mixed in two stages. In the first stage, EPDM was mixed with CB, HS, stearic acid, zinc oxide, and the flame retardants. CB acts as a reinforcing filler to receive appropriate mechanical properties. HS is an antiaging agent. Stearic acid and ZnO accelerate the vulcanization process by forming an intermediate complex. CZ and Thiuram MS are accelerators to improve the vulcanization process.

After vulcanization, CB, PANI, and the flame retardants exist as particles in the rubber composite, only. All other fillers reacted or changed during the vulcanization and were ether disturbed on a molecular level, chemically changed, or partially vaporized. The fillers with a significant influence on the mechanical properties are CB, PANI, APP, and ATH. The particle size of APP and ATH was more than one order of magnitude larger than the particle size of CB and PANI.

In the second stage, the curatives (Thiuram MS, CZ, and sulfur) were added. The first mixing stage took 4 min, the second 1 min, and afterward the rubber was 5 min on the rolls. The total mixing time of the compounds was 10 min. A dynamic moving die rheometer (D-MDR 300, Montech, Werkstoffprüfmaschinen, Buchen, Germany) was used to determine the curing time, at which 90% of the cross-linking took place (t_90_), and the minimum (ML), and maximum (MH) torque. The rubber composites were cured in a compression mold at a temperature of 160 °C and a pressure of 300 bar. For the characterization of the rubber composites, sheets with varying thicknesses of 2, 3, and 6 mm were prepared.

## 3. Characterization

A scanning electron microscope (Zeiss EVO MA 10, Zeiss, Oberkochen, Germany) with an acceleration voltage of 10 kV was used for the scanning electron microscopy (SEM) micrographs. The rubber samples were freeze-fractured and a gold-coated surface applied to the EPDM rubber composites.

The MCR 501 rheometer (Anton Paar, Graz, Austria) was used to determine the storage modulus (G’) and dynamic loss factor (tan δ) as a function of temperature at 1 Hz and 0.1% strain amplitude. The temperature ranged from 80 °C to 70 °C at a heating rate of 1 °C/min.

In accordance with ISO 37, the tensile tests were performed using five dumbbell samples of 2 mm thickness. Young’s modulus, yield stress, and elongation at break were determined on a stress–strain machine (Zwick/Roell Z010 instrument, Zwick, Ulm, Germany), using three dumbbell samples of 2 mm thickness, according to ISO 527. Shore A hardness was determined with three samples of 6 mm thickness according to ISO 7619-1.

Thermal conductivity was measured with a TPS 1500 (hot disk, Gothenburg, Sweden). In accordance with ISO 22007-4, the hot disk sensor was placed between two samples with a thickness of 6 mm.

The limiting oxygen index (LOI) was measured using a Stanton Redcroft instrument (Thermal Scientific, Mansfield, MA, USA) according to ISO 4589-2 (specimen size 112 × 6.5 × 3 mm^3^). UL 94-V and UL 94-HB measurements were performed according to IEC 60695-11-10 (specimen size 125 × 13 × 3 mm^3^). Cone calorimeter tests were performed according to ISO 5660 (specimen size 100 × 100 × 3 mm^3^) with a cone calorimeter (FTT, East Grinstead, UK). The samples were placed in a metal wire cage made from a 241 × 101 mm sheet of steel (1 mm wire with 9 mm mesh size) and measured using a retainer frame, whereas the ISO 5660 standard stipulates a wire cage without a frame [45]. The samples were tested with a distance of 35 mm between sample and burner and a heat flux of 50 kW·m^−2^, without any significant change to the heat flux distribution on the surface [46]. All measurements were done in duplicate and the results were averaged. If needed, an additional third measurement was carried out to clarify the results.

Glow wire tests were carried out according to DIN EN 60695-2. The tested samples were 60 × 60 × 3 mm^3^ in size. The glow wire test was performed to assess the probable applications of EPDM rubber compounds. The glow wire flammability index (GWFI) is the highest temperature at which no flame occurred, or at which flameout occurred within 30 s after the removal of the glowing wire. Additionally, the underlying paper had to be intact [47]. The glow wire ignition temperature (GWIT) is defined as the temperature that is 25 °C higher than the maximum temperature of the tip of the glow wire, which does not cause ignition of the material during three subsequent tests [47].

The FMVSS 302 test was performed according to DIN 75200. According to the standard, the tests were carried out on five samples with specimens 160 × 100 × 3 mm^3^ in size.

## 4. Results and Discussion

### 4.1. Curing Properties

**Figure 1:**
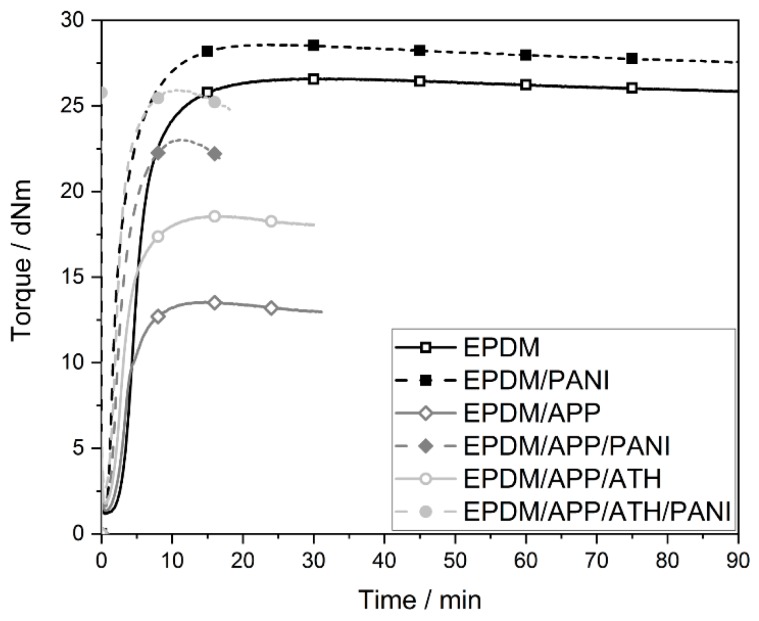
Torque over time of the EPDM rubber compounds. The addition of polyaniline (PANI) to the corresponding rubber compound is symbolized by the dashed lines.

The curing curves of the EPDM rubbers are shown in Figure 1. Vulcanization of the rubbers resulted in an increase in torque. Comparing EPDM and EPDM/PANI, the addition of PANI led to a higher torque, caused by the reinforcement of PANI. Because of the different polarities of PANI and EPDM, and due to results from former studies, it is assumed that PANI was not dispersed on a molecular level, but instead formed small lumps that were well dispersed in the EPDM matrix [48,49].

The incorporation of APP in EPDM/APP led to much lower torque, i.e., a reduction of about 50%, followed by reversion of torque, as seen in Figure 1 and Table 2. The reversion in torque is the result of cleaved sulfide cross-links, which result from extended curing. The addition of APP led to a reduction in the cross-linking in the rubber compound and showed a plasticizing effect. Furthermore, the adhesion of uncoated APP to EPDM is poor [50]. Therefore, the rigid APP particles had no reinforcing effect. The addition of PANI to EPDM/APP in EPDM/APP/PANI partially compensated for the plasticizing effect of APP. This compensation is explained by the fact that PANI has a rigid structure and therefore acted as a reinforcing filler.

The shape of the vulcanization curve of EPDM/APP/ATH looked like EPDM/APP, but with higher torque values. The increase in torque resulted from the high amounts of ATH, a rigid particle. This effect is known for rubbers with mineral fillers [10]. The vulcanization curve of the multicomponent rubber compound EPDM/APP/ATH/PANI was like the curve of EPDM/APP/PANI with increased torque values. The addition of PANI to the EPDM/APP/ATH rubber composite showed the same increase in torque as in the samples without ATH, EPDM/APP and EPDM/APP/PANI. As a result, the EPDM/APP/ATH/PANI compound exhibited the same torque values as the unfilled EPDM rubber compound and a faster vulcanization process.

The characteristic curing properties of the EPDM compounds are displayed in Table 2. All filled rubbers showed a shorter scorch time than the unfilled EPDM rubber. EPDM/PANI exhibited the lowest value of all rubbers because the reactive species were formed more quickly, and therefore the vulcanization started almost three times earlier. It is known that the presence of amines accelerates sulfur curing and shortens the scorch time [51,52,53]. In general, the rubber compounds containing PANI had a lower scorch time than the corresponding rubbers without this additive. The t_90_ represents the time needed to reach 90% of the maximum torque, and quantifies the time for the complete vulcanization of the rubber compounds. EPDM reached t_90_ at 9.77 min. All filled rubbers except EPDM/APP/ATH experienced a reduction of t_90_, as seen in Table 2.

Table 2 shows the minimum torque (ML), which is a measurement of the viscosity of the uncured rubber compound [54]. In general, the viscosity of the rubber increased with higher filler loading, therefore the addition of the reinforcing fillers resulted in higher values for the ML [55,56]. This effect occurred for all filled EPDM rubber compounds. The addition of PANI, APP, and ATH increased the ML. The rubber samples containing PANI showed higher values for ML than the corresponding samples without PANI. The compounds EPDM/APP/PANI and EPDM/APP/ATH showed similar ML values, indicating that 7 phr of PANI have the same effect on viscosity as 50 phr ATH. This effect highlights the reinforcing character of PANI and is explained by the smaller particle size of PANI. Fillers, and especially nano-fillers with higher aspect ratios, result in a higher impact on viscosity [57].

The maximum of torque (MH) is a measure of the stock modulus [56]. The EPDM/PANI rubber showed an MH value 10% higher than EPDM. The EPDM/APP had the lowest MH, experiencing a reduction of about 50% compared to EPDM. This reduction was partially compensated by PANI. EPDM/APP/PANI experienced a reduction in MH of 12% compared to EPDM. The reduction of MH was caused by the poor adhesion of APP to the EPDM polymer and the reduced crosslink density. The addition of ATH to EPDM/APP, resulting in EPDM/APP/ATH, led to a higher MH. The multicomponent rubber composite EPDM/APP/ATH/PANI exhibited MH values like those of the unfilled EPDM rubber.

The rheometer curves of the rubber compounds were used to describe the microstructural characteristics of the rubbers and reveal some physical parameters. MH and ML were used to assess the crosslink density of the EPDM compounds. MH–ML is a qualitative assessment of the crosslink density of a rubber vulcanizate [55]. The variation in the torque difference corresponds with the crosslink density values of the Flory–Rehner model. Therefore, the percentages of the MH–ML values decreased with reduced network density, because of increased slippage between the polymer chains [43]. It has been reported that the addition of higher amounts of modified PANI resulted in a decrease in cross-linking. This effect was caused by the acidic nature of the modified PANI, which inhibited the formation of free radicals for the vulcanization process [30,58]. This effect was not observed for the EPDM compounds in this study that contained PANI. The reduced torque (50%) for the EPDM/APP rubber, as seen in Figure 1, is explained by reduced crosslink density. The curing properties revealed that PANI acted as a reinforcing filler and compensated for most of the plasticizing effect in EPDM/APP/PANI, as was reflected by the increased MH–ML. The multicomponent rubber composite EPDM/APP/ATH/PANI exhibited a similar crosslink density as the unfilled EPDM rubber.

### 4.2. Scanning Electron Microscopy (SEM)

**Figure 2:**
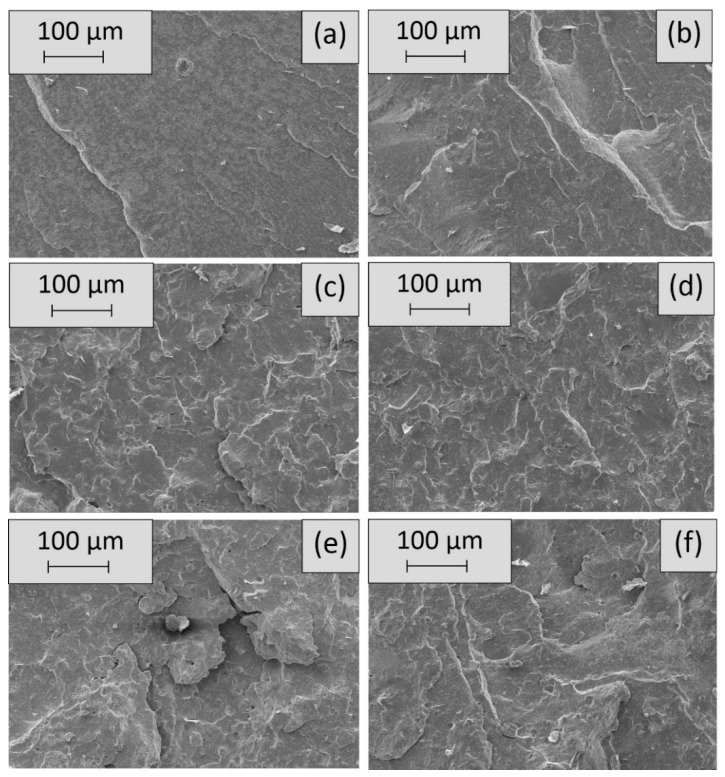
Scanning electron microscopy (SEM) micrographs of the rubbers EPDM (**a**), EPDM/PANI (**b**), EPDM/ammonium polyphosphate (APP) (**c**), EPDM/APP/PANI (**d**), EPDM/APP/ATH (**e**), EPDM/APP/aluminum trihydroxide (ATH)/PANI (**f**).

The SEM micrographs of the freeze-fractured surfaces of the EPDM compounds are displayed in Figure 2. Figure 2a shows the unfilled rubber (EPDM), which had the smoothest surface of all rubber samples because it had the lowest amount of fillers—no APP, PANI, or ATH. The addition of PANI, resulting in EPDM/PANI, led to an increased surface roughness because of the reinforcing effect of PANI, and thus contributes to the good interaction between filler and matrix, as seen in Figure 2b. The EPDM/APP and EPDM/APP/PANI compounds had a similar roughness and did not reveal significant differences between each other. When EPDM/APP and EPDM/APP/PANI were compared with EPDM and EPDM/PANI, an increase in roughness was detected. For all samples, no agglomerates were detected; therefore, the fillers were well dispersed in the rubber matrix. The rubber compounds containing ATH, EPDM/APP/ATH and EPDM/APP/ATH/PANI, were the two roughest samples and exhibited the highest protuberances. Rubber compounds with high filling grades of mineral fillers like ATH that show good filler-rubber interaction become stiffer and more brittle [59]. The particles (approx. 10 μm in size) in Figure 2c–f are attributed to the flame retardants APP and ATH [60].

### 4.3. Mechanical Properties

**Figure 3:**
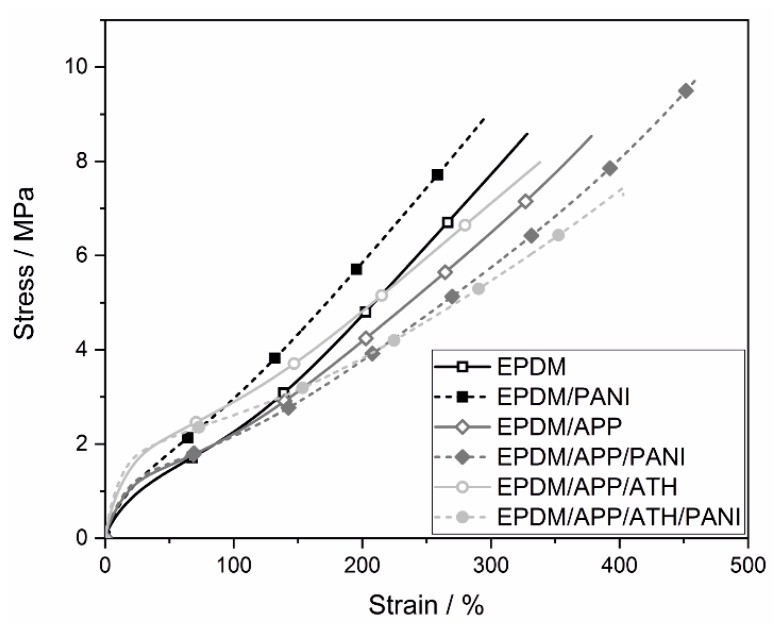
Stress–strain diagram of the EPDM rubbers.

The results of the mechanical tests of the EPDM compounds are presented in Figure 3 and Table 3. The EPDM rubber exhibited an elongation at break of 330%, as did EPDM/PANI. The impact of PANI on mechanical properties like elongation at break depends on the concentration of PANI in the rubber compound [48]. The EPDM/PANI rubber compound contained 5 wt.% of PANI. According to a study by Chen, the most effective loading of PANI to obtain improved mechanical properties was 3 wt.% [48]. Therefore, the amount of PANI in EPDM/PANI might be too high to achieve a better result for elongation at break. Furthermore, the formation of PANI lumps in the rubber compound, due to poor dispersion caused by the different polarities of PANI and the EPDM rubber, may also be detrimental [48,49]. Dispersion means more than mixing and distributing particles in the rubber matrix. Good dispersion often requires an energy-intensive process to achieve the breakdown of agglomerates, create large surfaces, and dispense PANI on the molecular level with good filler–matrix interaction, so that every particle is coated with rubber [49]. EPDM/APP showed a higher elongation at break of 378%, which corresponds to an increase of 15%. This increased elongation at break is explained by the lower cross-linking density of the rubber, as shown in Table 2. With less cross-linking in the rubber compound, the polymeric rubber chains gained enhanced flexibility. The rubber EPDM/APP/PANI experienced an even greater increase than EPDM/APP in elongation at break, of up to 446%. The amount of PANI in the EPDM/APP/PANI was 4 wt.% and resulted in an elongation at break 18% that was greater than EPDM/APP. The EPDM/APP/ATH exhibited an elongation at break equal to that of the unfilled EPDM. Two counteracting effects occurred, which balanced each other out. The addition of high amounts of mineral fillers made the material more brittle, while the reduced crosslink density made for a more flexible rubber compound. This shows that the combination of APP and ATH preserved the mechanical properties of the rubber compound and simultaneously increased its flame retardancy. Often, mechanical properties deteriorate with high loadings of mineral fillers [12]. The addition of PANI in EPDM/APP/ATH/PANI led to a 30% increase over EPDM/APP/ATH. This was the greatest increase in elongation at break found for the addition of PANI. The EPDM/APP/ATH/PANI compound contained 3 wt.% of PANI. For thermoplastic natural rubber (TPNR) compounds, the greatest increase in elongation at break occurred at a loading of around 3 wt.% [48]. The addition of PANI might potentially improve the adhesion of filler and matrix and result in higher elongation at break.

With a value of 6.7 MPa, the unfilled rubber, EPDM, had the lowest Young’s modulus of all samples, as seen in Table 3. This is due to the fact that the unfilled EPDM rubber had the lowest amount of reinforcing fillers, namely, only CB. The addition of PANI in EPDM/PANI led to an increased Young’s modulus of about 8.9 MPa (+2.2 MPa, +25%). PANI, as a rigid polymer, acted as a reinforcing filler [49]. The samples EPDM/APP and EPDM/APP/PANI experienced a further increase to about 10.0 MPa (+3.3 MPa, +50%). The APP, added as small and stiff particles, was well dispersed in the rubber matrix with no specific orientation, as seen in Figure 2, and contributed to the overall filler loading by 13 wt.%. The additional filler increased the stress transfer from the rubber matrix to the filler and led to an increase in the Young’s modulus. The addition of PANI to EPDM/APP to produce EPDM/APP/PANI did not reveal significant changes in the Young’s modulus. The addition of ATH resulted in the highest Young’s modulus, 23.2 MPa (+16.5 MPa, +250%) and 28.5 MPa (+21.7 MPa, +320%). This effect is caused by the high amounts of the stiff fillers APP and ATH. With increasing filler content, the filler–filler interaction rises, and inter-aggregate distances become smaller. Therefore, the formation of a filler network is more likely [61]. The incorporation of PANI in EPDM/APP/ATH/PANI resulted in a Young’s modulus 30% higher than for EPDM/APP/ATH, because more rigid and better dispersed filler was in the rubber compound. Since APP and ATH are round, stiff particles equal in size and well dispersed in the matrix, other factors such as the orientation and shape of the fillers, as well as their aspect ratio, did not influence the mechanical properties of the rubber compound significantly.

Table 3 summarizes the tensile strength of the EPDM rubber composites. The stress–strain curves are displayed in Figure 3. All rubber mixtures except EPDM/PANI showed tensile strength similar to the unfilled EPDM mixture, about 8–9 MPa. The EPDM/PANI compound experienced a 15% increase in tensile strength, to 10 MPa. The reinforcing effect of PANI in the EPDM/PANI sample was greater, because the EPDM and EPDM/PANI rubber samples contained less filler (no APP or ATH) than all of the other rubber compounds. Therefore, a filler network of rigid PANI was more likely. The cross-linking undergone by PANI made the material more stress resistant [49]. This effect was lost with the addition of APP and ATH. Tensile strength and elongation at break depend on the adhesion between filler and matrix. If the adhesion is not good, the failure originates in the interfacial region between the filler and rubber matrix [49].

The EPDM rubber exhibited stress of 2.26 MPa at 100% elongation, as seen in Table 3. The addition of PANI in EPDM/PANI resulted in higher stress of about 3 MPa, an increase of 30%, because of an intact filler network. The samples EPDM/APP and EPDM/APP/PANI exhibited similar values as the unfilled EPDM compound. Two effects were considered. The reduced cross-linking decreases the stress at 100% elongation, whereas the addition of rigid fillers counteracts this reduction. As seen in Figure 3, the curves of EPDM, EPDM/APP, and EPDM/APP/PANI are the same until 100% elongation. With further elongation, EPDM/APP and EPDM/APP/PANI exhibited lower stress than the unfilled EPDM, because the filler network collapsed. Furthermore, adhesion of uncoated APP in EPDM was poor, and thus responsible for the deterioration of mechanical properties [50]. EPDM/APP/ATH and EPDM/APP/ATH/PANI showed stress of 2.6–2.9 MPa, a 30% increase in stress compared to EPDM, which is a result of the high loading of ATH.

The hardness showed the same pattern as the Young’s modulus. The reinforcement is proportional to stiffness, and therefore to the mechanical properties of the rubber materials. EPDM had a hardness of about 67 Shore A. The EPDM/PANI, EPDM/APP, and EPDM/APP/PANI compounds exhibited a minor increase in hardness, with values of about 69 Shore A, as seen in Table 3. The composites containing ATH, EPDM/APP/ATH and EPDM/APP/ATH/PANI, exhibited hardnesses of over 76 Shore A. This additional increase was caused by the higher amount of rigid fillers in the compounds [8].

### 4.4. Dynamic Mechanical Properties

**Figure 4:**
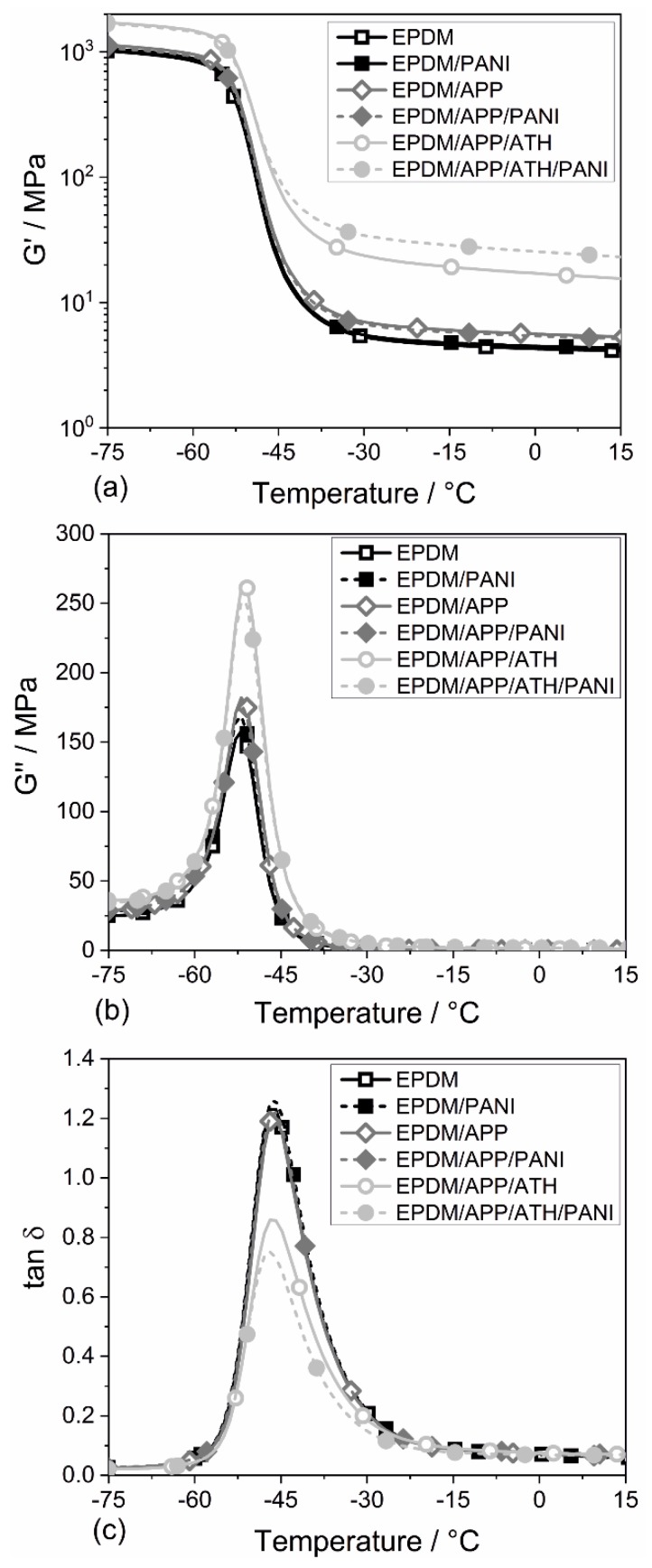
Storage modulus (G’) (**a**), loss modulus (G’’) (**b**), and loss factor (tanδ) (**c**) as a function of temperature of all EPDM rubbers.

Figure 4 presents the results of the dynamic mechanical analysis (DMA) of the EPDM rubber compounds. Figure 4a displays the storage modulus (G’) as a function of temperature. G’ is related to the stiffness of the rubber. Therefore, the changes among the rubber compounds are more pronounced above the glass transition temperature (*T*_g_), because the rubber itself is less stiff. In other words, there is a greater difference in stiffness between the filler and the matrix. The dynamic of immobilization of the rubber layers and the stress transfer to the filler are the main influences on the stiffness of rubber. EPDM and EPDM/PANI showed similar curves. EPDM/APP and EPDM/APP/PANI showed no differences between each other, but a minor increase in G’ compared to EPDM. This is due to the addition of rigid fillers. The addition of ATH led to the strong increase in G’. The EPDM/APP/ATH/PANI sample showed an even higher G’ than EPDM/APP/ATH. The EPDM/APP/ATH/PANI sample is the only sample containing PANI that exhibited higher values for G’ above the *T*_g_ than the corresponding PANI-free sample. This is worth noting because the EPDM/APP/ATH/PANI sample had the highest amount of filler and therefore the lowest percentage of PANI loading, but showed the greatest changes. The results of G’ and the Young’s modulus correspond well with each other.

Figure 4b displays the loss modulus (G’’) as a function of temperature of all the EPDM rubbers. EPDM showed the lowest maximum value for G’’. The addition of PANI in EPDM/PANI increased the G’’. The corresponding rubbers containing APP, EPDM/APP and EPDM/APP/PANI, experienced a further increase in G’’, but both compounds showed the same value. The higher maximum value of G’’ indicated that more energy was dissipated as heat. The samples containing ATH showed the highest values of G’’, but EPDM/APP/ATH/PANI had a lower value than the corresponding PANI-free sample EPDM/APP/ATH.

The loss factor (tanδ), as a function of temperature of the EPDM rubbers is shown in Figure 4c. At lower temperatures, the tanδ values were low because the viscosity of the rubber was high, suggesting hardly any movement of the polymer segments. The fillers had no evident effect on the energy dissipation [62]. All EPDM compounds showed the same peak of the loss factor at around −45 °C, indicating the α-transition of EPDM at *T*_g_ [31]. All rubber compounds showed a similar *T*_g_. The unchanged peak of tanδ in EPDM/APP and EPDM/APP/PANI indicates that APP exhibited poor filler–rubber interactions, which corresponds well with the results of the mechanical test. The rubber containing ATH, EPDM/APP/ATH, showed a prominent reduction in the peak of the loss factor. Furthermore, the value for EPDM/APP/ATH/PANI was 10% lower than for the EPDM/APP/ATH rubber compound. This effect is explained by the fact that the rubbers containing ATH exhibited more rubber–filler interaction, which restricted the motion of the rubber chains and resulted in a reduced tanδ [62]. High values of tanδ indicate that the material has a higher nonelastic strain component, so that with lower values of tanδ the rubber becomes more elastic. At higher temperatures the rubber molecules adjust quickly enough to follow the dynamic strain, because the viscosity becomes so low that the thermal energy is comparable to the potential energy barriers to rotation of the rubber segments [62].

### 4.5. Thermogravimetric Analysis

**Figure 5:**
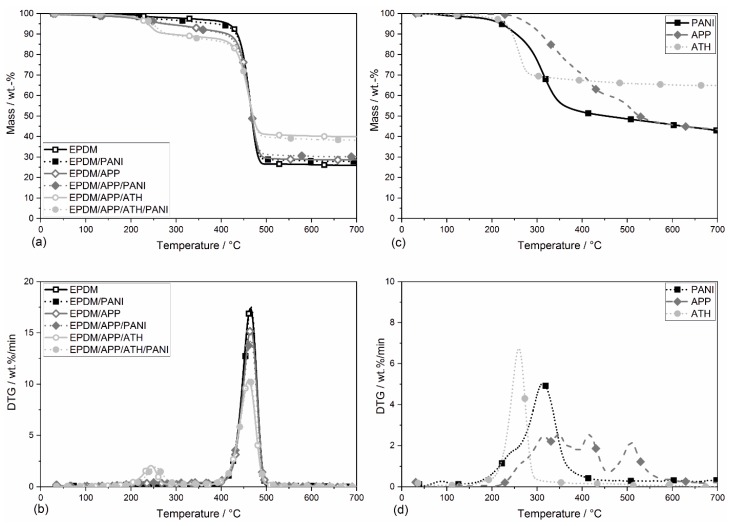
Mass loss (**a**) and mass loss rate (**b**) of the EPDM rubber compounds. Mass loss (**c**) and mass loss rate (**d**) of the flame retardants over temperature.

The results of the thermogravimetric analysis (TGA) are presented in Figure 5 and Table 4. The unfilled EPDM rubber showed a single decomposition step. For EPDM, thermal decomposition, the temperature at which 5% of the total mass is lost (*T*_5 wt.%_), started at 412 °C. The maximum of the mass loss rate, (*T*_max_) of EPDM was at 464 °C. During the main decomposition step of the rubber compound, the EPDM polymer decomposed and released volatile hydrocarbons formed by free radicals [63]. At a temperature of 600 °C the EPDM rubber had a residue of 24.6 wt.%. The EPDM/PANI compound decomposed at 389 °C and showed an increase in residue of about 15%, as seen in Figure 5. One way to assess the char-enhancing effect of flame retardant systems is to compare the calculated residue with the measured residue. Therefore, TGA analysis of all flame retardants was carried out and their residues at 600 °C were determined, as seen in Figure 5 and Table 4.

The measured residue was 28.0 wt.%, and the calculated residue was 2.5 wt.% lower at 25.5 wt.%. The residue was calculated by adding the proportional residues of the components, rubber, and flame retardants. EPDM/PANI consisted of 0.95 wt.% of the EPDM compound and 5 wt.% of PANI, e.g., (0.95 × 24.6) wt.% + (0.05 × 43.9) wt.% yielded a calculated residue of 25.6 wt.%, as seen in Table 4. The fact that the measured residue was higher than the calculated residue, showed that PANI acted as a char promoter. Former studies executed by Feaz et al. showed a decrease in the temperature of the main decomposition step for peroxide cured EPDM rubber compounds containing PANI [30]. The sulfur-cured EPDM rubber compounds used in this study did not show this effect. *T*_max_ was similar for all tested EPDM compounds. The main decomposition step was dominated by the polymer matrix. Because all EPDM rubbers had the same polymer matrix, *T*_max_ remained unchanged.

All rubber compounds containing APP exhibited an earlier start of decomposition. EPDM/APP and EPDM/APP/PANI decomposed at 210 °C and 215 °C, EPDM/APP/ATH and EPDM/APP/ATH/PANI at 238 °C and 248 °C, respectively. EPDM/APP and EPDM/APP/PANI decomposed slowly until the polymer matrix decomposed in the main decomposition step, when the maximum rate is reached. Addition of APP resulted in an increased residue for EPDM/APP (16%) and EPDM/APP/PANI (23%), compared to EPDM. Table 5 summarizes the measured and the calculated residues as well as the proportion of the rubber components in the residue. The calculated residue and measured residue for EPDM/APP were the same within the margin of error. EPDM/APP/PANI showed a small difference between the calculated and the measured residue, suggesting a minor char-enhancing effect.

The rubbers containing ATH, EPDM/APP/ATH and EPDM/APP/ATH/PANI, had two distinct decomposition steps. As seen in Figure 5d, the first decomposition step at around 250 °C, a mass loss of about 8 wt.%, corresponds with the decomposition of ATH in the rubbers [64]. The addition of ATH partially compensated for the reduction in *T*_5 wt.%_ of APP. The two rubber composites EPDM/APP/ATH and EPDM/APP/ATH/PANI exhibited an increase in residue of 63% and 57% compared to EPDM. The calculated residue of EPDM/APP/ATH and EPDM/APP/ATH/PANI was similar. The addition of PANI led to no further improvement in terms of residue formation. Nevertheless, both compounds containing ATH had more residue measured than was calculated, as seen in Table 5.

### 4.6. Thermal Conductivity and Heat Capacity

Thermal conductivity occurs via phonon (atomic vibration) movement through the polymeric matrix and the fillers [65]. It is well known that the dissipation of heat is of great importance for maximizing the lifespan of high-performance devices [66]. Furthermore, it has been seen that materials with higher thermal conductivity perform better in terms of flammability [67]. The thermal conductivity and the heat capacity of the EPDM rubbers are presented in Table 6. EPDM had a thermal conductivity of 0.29 W·m^−1^·K^−1^. The incorporation of PANI and APP left the thermal conductivity of the EPDM compounds unchanged. The addition of ATH resulted in an increase in the thermal conductivity of 50% over EPDM, to 0.44 W·m^−1^·K^−1^. This effect was explained by the high amounts of ATH (50 phr) applied in the rubber composites and the higher thermal conductivity of ATH, compared to the polymer matrix. A different trend occurred for the heat capacity of the EPDM rubbers. The unfilled EPDM rubber showed a heat capacity of 1.71 MJ·m^−3^·K^−1^. All other rubber compounds had similar values for heat capacity.

### 4.7. Flammability

The evaluation of the flammability of the EPDM rubbers in terms of reaction to small flame was investigated in the LOI test and UL 94 tests, as seen in Table 7. The unfilled EPDM rubber had an LOI value of 20.5 vol%. The incorporation of PANI in EPDM/PANI resulted in an LOI increased to 22.2 vol%. EPDM/APP exhibited an increase in LOI to 25.0 vol%. The combination of APP and PANI in EPDM/APP/PANI led to no further improvement over EPDM/APP. The addition of 50 phr ATH in EPDM/APP/ATH left the LOI unchanged compared to EPDM/APP. In contrast, the addition of PANI in EPDM/APP/ATH/PANI showed a further increase in the LOI to 26.6 vol%.

The results of the glow wire test are summarized in Table 7. The EPDM rubbers showed glow wire ignition temperature (GWIT) values of 700–825 °C and glow wire flammability index (GWFI) values of 775–960 °C. The EPDM/PANI sample exhibited an increased GWIT value of 750 °C (+50 °C) compared to the unfilled EPDM rubber. The addition of APP in EPDM/APP and EPDM/APP/PANI left the performance in terms of GWIT unchanged compared to EPDM. The incorporation of ATH in EPDM/APP/ATH and EPDM/APP/ATH/PANI led to the highest observed GWIT values, 800 °C (+100 °C) and 825 °C (125 °C). As seen in Table 7, the variation in the GWFI values of the EPDM rubbers is more distinctive than in the GWIT values. EPDM had a value of 775 °C. The addition of PANI to produce EPDM/PANI resulted in a higher GWFI value of 800 °C (+25 °C). The corresponding rubbers containing APP, EPDM/APP and EPDM/APP/PANI, showed a further increase in GWFI to 900 °C (+125 °C) and 960 °C (+185 °C). The two rubbers containing ATH, EPDM/APP/ATH and EPDM/APP/ATH/PANI, also showed the best performance, with GWFI values of 960 °C (+185 °C).

The UL 94 test was carried out on specimens with a thickness of 3 mm. The results of all EPDM compounds are presented in Table 7. The addition of the flame retardants APP and ATH as well as PANI left the UL 94 classification unchanged. All samples achieved HB classification in the UL 94 test. To obtain a better understanding of the flammability of the rubbers, the burning rates of the EPDM compounds measured in the UL 94 test were compared with the burning rates from the FMVSS 302 test. The unfilled EPDM rubber showed the highest burning rate among all rubbers in both tests. The addition of PANI in EPDM/PANI resulted in a burning rate reduced by 30% in the UL 94 test and by 50% in the FMVSS 302 test. The addition of APP, producing EPDM/APP and EPDM/APP/PANI, reduced the burning rate in the UL 94 test significantly. In contrast to the other rubbers, EPDM/APP/PANI exhibited a higher burning rate than the corresponding PANI-free rubber. The samples containing ATH showed low burning rates, with the EPDM/APP/ATH/PANI sample performing best. In the FMVSS 302 and UL 94, test specimens that experienced sample self-extinguishment are classified with a burning rate of zero. These results show that the multicomponent system consisting of a phosphorous flame retardant APP, the mineral filler ATH, and the synergist PANI achieved the best performance in this study, thus constituting a promising approach to obtain V classification for EPDM rubbers.

### 4.8. Fire Behavior

The ignitability and burning behavior of the EPDM compounds in a well ventilated developing fire scenario was determined in the cone calorimeter test, and the results are shown in Table 8. The ignition of the polymers depends mainly on the thermal conductivity, heat absorption, and thermal inertia, which is the product of material density and heat capacity [62]. Because the heat capacity and thermal conductivity of the filled EPDM rubbers are similar, the time to ignition for the rubbers did not vary much, either. The EPDM had a time to ignition (t_ig_) of 50 s; see Table 8. Addition of PANI decreased the time to ignition by 5 s. The addition of APP, to produce EPDM/APP and EPDM/APP/PANI, resulted in ignition 43 s and 37 s earlier. The rubbers containing PANI ignited earlier than the corresponding PANI-free compounds. The rubber compounds with ATH added exhibited the same time to ignition as the unfilled EPDM rubber. It is known that many systems containing flame retardants start to decompose earlier than their corresponding flame-retardant free polymers. This often leads to earlier ignition of the flame retarded polymer compared to the non-flame retarded polymer [42].

**Figure 6:**
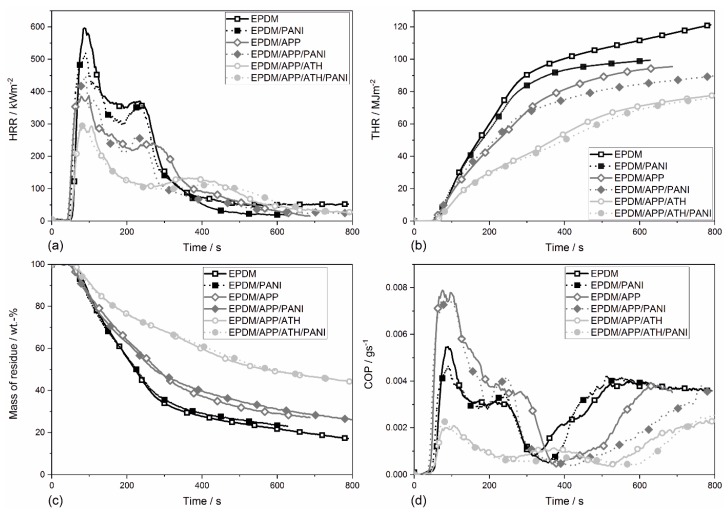
Heat release rate (HRR) curves (**a**), total heat release rate (THR) curves (**b**), mass of residue (**c**) and the carbon monoxide production (COP) (**d**) over time of the EPDM rubber compounds.

Figure 6a presents the heat release rate (HRR) curves of the EPDM rubbers. All of the rubber compounds showed the typical curve shape for thermally thick charring materials with poor protective properties [68]. After ignition, the HRR of the rubber compounds showed a first peak, followed by a second lower and broader peak. In general, the rubbers containing APP and ATH showed lower values for HRR than the unfilled EPDM rubber. APP formed a protective layer which limited the release of the volatiles, because the protective layer acted as a heat shield. This mode of action is well known for phosphorous flame retardants [44,69]. The second peak results from the thermal feedback when the pyrolysis front reaches the back of the specimen [70]. Additionally, cracking of the protective layer is a reasonable explanation for the second peak [68].

The EPDM rubber showed a peak of heat release rate (PHRR) of 598 kW·m^−2^. The addition of PANI reduced the PHRR to 531 kW·m^−2^ (−67 kW·m^−2^, 11%). EPDM/APP and EPDM/APP/PANI showed a further reduction in PHRR, to 399 kW·m^−2^ (−199 kW·m^−2^, 33%) and 440 kW·m^−2^ (−158 kW·m^−2^, 26%), respectively. The incorporation of PANI in the EPDM compound containing APP led to no further reduction of the PHRR. Instead, the EPDM/APP/PANI had a PHRR 11% higher than EPDM/APP. A different trend was observed for the two rubber composites containing ATH, EPDM/APP/ATH and EPDM/APP/ATH/PANI. These two composites exhibited equal values for PHRR. One reasonable explanation for the limited impact of PANI in the compounds containing ATH is that the percentage of PANI in the rubber composites containing ATH is lower than in the ATH-free rubber compounds. Nevertheless, the compounds containing ATH achieved the lowest values for PHRR.

The total heat release rate (THR) over time of the EPDM compounds is presented in Figure 6b. The unfilled EPDM rubber showed the highest THR curve of all rubbers. The addition of PANI led to a reduced THR. The same trend was observed for the EPDM/APP and EPDM/APP/PANI compounds. This effect was caused by fuel replacement and is not contributed to the synergist effects. The most prominent reduction in THR was achieved by the addition of ATH. This strong reduction is explained by the high ATH (50 phr) content used. These high loadings of ATH led to fuel dilution in the condensed phase. Since the THR curve of EPDM/APP/ATH/PANI is slightly lower than the THR curve of EPDM/APP/ATH, the addition of PANI had only a minor impact on the THR. The addition of PANI to the ATH-containing rubbers had only limited impact, because the relative amount of PANI was much smaller. Therefore, the effect of fuel replacement was little. The THR at flameout is the total heat evolved (THE) and measures the fire load of the specimen in the cone calorimeter fire scenario [68]. The THE values of all EPDM rubbers are presented in Table 8. Comparison of the rubbers containing PANI with the corresponding PANI-free rubbers suggests that the reduction in THE was caused mainly by the flame retardants APP and ATH.

The same trend was observed for the changes in amount of residue formed, as seen in Figure 6c and Table 8. The rubber EPDM exhibited a residue at flameout of 30 wt.%. The residue of the EPDM compounds resulted mainly from the reinforcing filler carbon black. Under anaerobic conditions these carbon fillers are thermally stable and therefore do not decompose during the pyrolysis phase. The anaerobic decomposition occurred while the rubber was burning with a stable flame [71]. The amount of residue left at the end of combustion is a parameter to quantify the condensed phase activity of flame retardants. EPDM/PANI had 2% more residue at flameout than the unfilled EPDM rubber. The increase in residue of the rubbers EPDM/APP and EPDM/APP/PANI to 40 wt.%. In the TGA the EPDM/APP/PANI compound showed a little more residue than the EPDM/APP. One reason, why the PANI-containing rubbers sometimes exhibited less residue than the corresponding PANI-free samples was that the flameout of the PANI-containing rubber was much later. Therefore, more material decomposed during the additional time. Secondly, the cone calorimeter test, in contrast to TGA, is a real fire test, which also determined protective layer effects, for example. Nevertheless, the results of both experiments correspond well with each other, within the margin of error. The two composites containing ATH showed 50 wt.% residue, resulted from the flame retardants APP and ATH. These results correspond well with the conclusions from TGA tests. The more fuel is fixed in the condensed phase, the less fuel contributes to the THE. Therefore, the increased residue caused a reduction in THE. In the TGA test, EPDM/APP/PANI exhibited increased residue compared to EPDM/APP. This effect was not found in the cone calorimeter test. The rubber samples containing ATH showed the same trend as in the TGA test.

Table 8 displays the effective heat of combustion (EHC) of the rubbers. EHC is an important parameter for the characterization of a material’s burning behavior. EHC is the quotient of the THE and the total mass loss, and is a parameter to quantify the gas activity [44]. The unfilled EPDM rubber had an EHC of 41.6 MJ·kg^−1^. The addition of PANI in EPDM/PANI reduced the EHC by 8%, to 38.0 MJ·kg^−1^. The addition of the flame retardants APP and ATH reduced the EHC further. The reduction occurred due to the release of water and nitrogenous volatiles, which caused flame dilution. In combination with APP, the compounds containing PANI exhibited a lower EHC than the corresponding PANI-free samples. But the difference in EHC decreased with increasing amount of filler.

The values for the total smoke release (TSR) of the EPDM rubbers are summarized in Table 8. The TSR of EPDM was 1562 m^2^·m^−2^. EPDM/PANI and EPDM/APP/PANI left the TSR values unchanged, whereas EPDM/APP showed a slight increase in TSR to 1795 m^2^·m^−2^. The composites containing ATH reduced the TSR by 50%. For the rubber compounds with flame retardants it was concluded that PANI acted as a smoke suppressor, because rubbers containing PANI showed lower TSR values than the corresponding PANI-free samples. A reasonable explanation is that the addition of PANI led to an improved char-forming process and therefore barrier effect, which led to reduced smoke production. This effect was also seen in the multicomponent system EPDM/APP/ATH/PANI, even though the concentration of PANI was only 3 wt.%.

After flameout, the rubbers underwent thermal oxidative decomposition, known as afterglow [71]. Figure 6d shows the carbon monoxide production (COP) curves of the EPDM rubber compounds. During pyrolysis, the shape of the COP curve is shaped like the HRR curve, with a double peak profile. Flameout of the rubber compounds is characterized by a minimum in the COP curve, as seen in Figure 6d. During afterglow, all flame retardant rubbers exhibited a reduced COP. This is known as afterglow suppression, and occurs due to an increased thermal stability at the top layer of the residue. The flame retarded rubbers containing PANI showed a small but significant reduction in COP compared to the corresponding PANI-free rubber samples. Again, this effect is caused by improved and thermally stable char. One explanation is that the addition of PANI led higher amounts incorporated nitrogen or P–N species in the residue.

The real flame spread above the surface of the specimen cannot be measured with a single parameter from the cone calorimeter without oversimplifying the scenario [72]. Therefore, various indices have been used to assess the hazard of developing fires under real conditions. Two widely used values are the fire growth rate (FIGRA) and the maximum average rate of heat emission (MARHE) [68,72]. The FIRGA and MARHE values of the rubbers are presented in Table 8. The unfilled EPDM rubber showed a FIGRA of 7.1 kW·m^−2^·s^−1^ and a MARHE of 314 kW·m^−2^. Compared to EPDM, EPDM/APP and EPDM/APP/ATH showed respective reductions of 20% and 50% in the FIGRA, and of 25% and 50% in the MARHE. The addition of PANI left the FIGRA values unchanged. The same applies to the MARHE, except for the composites containing ATH, i.e., EPDM/APP/ATH/PANI had a MARHE 10% lower than EPDM/APP/ATH, showing the best performance with a MARHE of 142 kW·m^−2^.

### 4.9. Phosphorous Flame Retardant Modes of Action

To receive a broader understanding of the phase activity, and therefore of the mode of action of the phosphorous flame retardants, elementary analysis of the rubbers containing rubbers APP was carried out. The phosphorus content in the rubber compounds and in the residues is summarized in Table 9. The residues were taken from the cone calorimeter and were quenched with liquid nitrogen at flameout. The differences in the P-content of the samples is explained by the different amounts of filler in the rubber. The amount of phosphorus in the residue was the same for EPDM/APP and EPDM/APP/PANI. The reduced P-content of EPDM/APP/ATH is explained by the additional residue formed. ATH decomposed to Al_2_O_3_, which remained in the condensed phase.

To determine the amount of phosphorus released into the gas phase, the amount of phosphorus in the residue was subtracted from the amount of phosphorus in the sample. For all four EPDM compounds containing APP, it was found that the complete amount of phosphorus remained in the residues of the rubbers. Therefore, APP showed condensed phase activity only, as seen in Table 9. The addition of PANI left the mode of action of APP unchanged.

Previous studies with various flame retarded polymeric materials quantified the effects of flame retardants [73,74,75]. The Equations (1) and (2) were used to calculate the theoretical values of PHRR and THE, with χ the combustion efficiency, μ the char yield, $h_{c}^{0}$ the heat of complete combustion of the fuel gases, and m_0_ the mass of the specimen.

(1) $$\mathrm{HRR}\;\sim \;\chi \cdot \left(1\;-\;\mu \right)\cdot h_{c}^{0}$$

(2) $$\mathrm{THE}\;\sim \;\chi \cdot \left(1\;-\;\mu \right)\cdot h_{c}^{0}\cdot m_{0}$$

The reduction in the EHC $\chi \cdot h_{c}^{0}$ is influenced by flame inhibition as well as fuel dilution, and this is displayed along with the effect of fuel reduction (1 − μ) and the altered density due to the addition of a flame retardants [73]. The value of THE is calculated by measuring the change in the sample weight, the formed residue (1 − μ), and the reduction in EHC (χ·$h_{c}^{0}$). The calculated THE and the measured THE are presented in Table 10. Both values are in good accordance to each other. The rubbers containing ATH show the greatest deviation, with around 9%. This deviation was caused by the change in density of the rubber compounds. The shown values are expressed as a percentage of the unfilled EPDM rubber, so values of EPDM are always set to 100%. In contrast to the calculated values of PHRR (using Equation (1)), the reduction in the measured PHRR includes the effect of the protective layer, which means that these two values of measured and calculated PHRR differ only in terms of this effect. The calculations of the protective layer effect in the EPDM/APP/ATH/PANI sample were carried out next. The EPDM/APP/ATH/PANI had a residue of 51 wt.% and the unfilled EPDM of 30 wt.%, as seen in Table 10. The increased residue of EPDM/APP/ATH/PANI resulted from the addition of APP, ATH, and PANI amounts to a total of 21 wt.%. Compared to the unfilled EPDM rubber, that led to a fuel reduction (1 − μ) of 79.0%. In the cone calorimeter test the EHC of the EPDM (41.6 MJ/kg) and the EPDM/APP/ATH/PANI (31.9 MJ/kg) were measured, and EPDM/APP/ATH/PANI showed a 23.3% reduction in the EHC. These two values were multiplied with each other (0.790 × 0.767), resulting in a calculated PHRR 60.5% that of the unfilled EPDM rubber. The measured PHRR of EPDM/APP/ATH/PANI was 49.2% that of the EPDM rubber. The additional reduction in the PHRR is a result of the protective layer effect [73]. The rubbers containing APP exhibited a fuel reduction of 10%; the compounds containing ATH had a reduction of over 20% compared to the unfilled EPDM rubber. The heat of combustion was reduced by about 15–25%. Theoretically, these two effects add up to a reduction in THE of 25–45%, but this reduction is compensated to some extent by the increased density of the rubber compounds caused by the additional flame retardants, resulting in a mass per specimen of about 10–30% higher than the unfilled EPDM rubber. Therefore, the overall reduction in the THE of the rubbers containing APP is in the range of 15–25%.

EPDM/PANI exhibited a small reduction in the THE, but no improvement in the protective layer effect compared to EPDM. The addition of the flame retardants APP and ATH in EPDM/APP/ATH and EPDM/APP/ATH increased the protective layer effect by 13.4% and 19.0%, respectively. In combination with PANI, the protective layer effect was reduced to 0.1% in EPDM/APP/PANI. Due to polarity, PANI and APP stayed together in the rubber matrix. Therefore, PANI might prevent APP from forming a protective layer. The two rubbers containing ATH, EPDM/APP/ATH and EPDM/APP/ATH/PANI, experienced similar protection layer effects.

## 5. Conclusions

This study investigated multicomponent EPDM rubbers by systematically varying the flame retardants APP and ATH, and the addition of the nitrogenous synergist PANI. Rheological measurements as well as SEM micrographs revealed that the flame retardants were well dispersed in the rubber matrix. The addition of the synergist PANI in the composite EPDM/APP/ATH/PANI showed that the addition of PANI improved mechanical properties, i.e., elongation at break, Young’s modulus, and tensile strength. TGA of EPDM/PANI and EPDM/APP/PANI showed that an increased residue yield was achieved by the incorporation of PANI. In the LOI test the multicomponent system with the synergist PANI EPDM/APP/ATH/PANI showed the highest LOI value. In the glow wire tests the rubber compounds containing PANI outperformed the corresponding PANI-free samples. The combination of the two flame retardants APP and ATH in combination with PANI in EPDM/APP/ATH/PANI showed the best performance in the glow wire test. The cone calorimeter test revealed that the addition of PANI decreased THE, EHC, and MARHE. APP and ATH combined with PANI achieved a reduction of more than 50% in MARHE, to 142 kW·m^−2^. Furthermore, the COP showed that PANI in combination with APP and ATH acted as an afterglow suppressor. All EPDM residues were analyzed via elementary analysis and it was shown that all of the phosphorus in APP remained in the condensed phase. Although the chemical structure of PANI indicates a possible application as a char precursor, the rubbers containing PANI showed no increased formation of protection layer for the flame retarded EPDM rubber compounds. Nevertheless, the strategy of using multicomponent EPDM rubbers containing flame retardant and the synergist PANI resulted in improved mechanical properties and increased flame retardancy.

## Figures and Tables

**Table 1:** Formulation of the ethylene propylene diene monomer rubber (EPDM) composites.

Fillers	EPDM	EPDM/PANI	EPDM/APP	EPDM/APP/PANI	EPDM/APP/ATH	EPDM/APP/ATH/PANI
EPDM	100	100	100	100	100	100
APP	-	-	21	21	21	21
PANI	-	7.0	-	7.0	-	7.0
ATH	-	-	-	-	50	50
Antioxidant	0.5	0.5	0.5	0.5	0.5	0.5
Zinc oxide	5.0	5.0	5.0	5.0	5.0	5.0
Stearic acid	1.0	1.0	1.0	1.0	1.0	1.0
CB	30	30	30	30	30	30
CZ	1.0	1.0	1.0	1.0	1.0	1.0
Thiuram MS	2.0	2.0	2.0	2.0	2.0	2.0
Sulfur	1.5	1.5	1.5	1.5	1.5	1.5

**Table 2:** Curing characteristics of the EPDM rubber compounds.

Parameters	EPDM	EPDM/PANI	EPDM/APP	EPDM/APP/PANI	EPDM/APP/ATH	EPDM/APP/ATH/PANI
Scorch time (±0.4)/min	2.88	0.88	1.88	1.14	1.43	1.21
t_90_ (±1.3)/min	9.77	7.74	7.11	6.07	6.65	5.04
MH (±1.5)/dN·m	26.6	28.6	13. 6	23.0	18.6	25.9
ML (±0.1)/dN·m	1.17	1.28	1.35	1.60	1.77	2.57
MH–ML	25.4	27.3	12.3	21.4	16.8	23.4
Variation in the MH–ML/%	100	107	48	84	66	92

**Table 3:** Mechanical properties of the EPDM compounds.

Parameter	EPDM	EPDM/PANI	EPDM/APP	EPDM/APP/PANI	EPDM/APP/ATH	EPDM/APP/ATH/PANI
Elongation at break (±21)/%	330	326	378	446	338	422
Young’s modulus (±1.0)/MPa	6.73	8.63	10.0	10.9	23.2	28.5
Tensile strength (±0.59)/MPa	8.61	9.91	8.51	9.12	8.18	7.86
Stress at 100% (±0.1)/MPa	2.26	2.97	2.16	2.01	2.89	2.60
Hardness (±0.6)/Shore A	66.9	69.9	68.3	69.0	76.3	77.1

**Table 4:** Thermogravimetric analysis (TGA) results of the EPDM rubbers. Temperature at which 5 wt.% of the mass was lost (*T*_5 wt.%_) and temperature of the maximum of the mass loss rate (*T*_DTGmax_) are presented. The residue of the rubber samples was determined at 600 °C.

Material	*T*_5 wt.%_ (±2)/°C	*T_DTG_*_(1)_ (±2)/°C	Mass Loss at *T_DTG_*_(1)_ (±0.5)/wt.%	*T*_DTGmax_ (±2)/°C	Residue (±1)/wt.%
PANI	212	-	-	312	43.9
APP	277	-	-	347	45.7
ATH	230	-	-	260	65.2
EPDM	412	-	-	464	24.6
EPDM/PANI	389	-	-	466	28.0
EPDM/APP	210	-	-	465	28.7
EPDM/APP/PANI	215	-	-	465	30.4
EPDM/APP/ATH	238	246	8.1	461	40.2
EPDM/APP/ATH/PANI	248	254	8.1	463	38.6

**Table 5:** Measured and calculated residues of the rubber compounds with the proportional contribution of the flame retardants. The calculated residues were based on the TG results and therefore carried out with a margin of error of about ±1 wt.%.

Rubber	Residue (±1)/wt.%	Calc. Residue	Residue of EPDM	Residue of PANI	Residue of APP	Residue of ATH
EPDM	24.6	-	24.6	0.0	0.0	0.0
EPDM/PANI	28.0	25.6	23.4	2.1	0.0	0.0
EPDM/APP	28.7	27.3	21.4	0.0	5.9	0.0
EPDM/APP/PANI	30.4	28.0	20.5	1.8	5.7	0.0
EPDM/APP/ATH	40.2	36.3	16.4	0.0	4.5	15.4
EPDM/APP/ATH/PANI	38.6	36.5	15.8	1.4	4.4	14.9

**Table 6:** Thermal conductivity and heat capacity of the EPDM rubber mixtures.

Parameter	EPDM	EPDM/PANI	EPDM/APP	EPDM/APP/PANI	EPDM/APP/ATH	EPDM/APP/ATH/PANI
Thermal conductivity (±0.01)/W·m^−1^·K^−1^	0.29	0.29	0.31	0.31	0.44	0.44
Heat capacity (±0.02)/MJ·m^−3^·K^−1^	1.71	1.74	1.75	1.76	1.75	1.74

**Table 7:** LOI, glow wire, and UL94 results of the EPDM rubbers.

Parameter	EPDM	EPDM/PANI	EPDM/APP	EPDM/APP/PANI	EPDM/APP/ATH	EPDM/APP/ATH/PANI
LOI (±0.2)/vol%	20.6	22.2	25.0	24.0	25.1	26.6
GWIT (±25)/°C	700	750	700	700	800	825
GWFI (±25)/°C	775	800	900	960	960	960
UL94	HB	HB	HB	HB	HB	HB
UL 94/mm·min^−1^	18.2	12.4	0.5	3.9	2.4	0
FMVSS 302/mm·min^−1^	27.5	13.8	0	0	0	0

**Table 8:** Cone calorimeter results of EPDM rubber composites with an irradiance of 50 kW·m^−2^.

Parameter	EPDM	EPDM/PANI	EPDM/APP	EPDM/APP/PANI	EPDM/APP/ATH	EPDM/APP/ATH/PANI
*t*_ig_ (±2)/s	50	45	43	37	51	48
PHRR (±5)/kW·m^−2^	598	531	399	440	295	294
Flameout (±5)/s	315	348	366	408	516	546
Residue (±1)/wt.%	30	32	40	39	52	51
TSR (±40)/m^2^·m^−2^	1562	1595	1795	1612	852	714
THE (±4)/MJ·m^−2^	93	90	78	76	65	65
EHC (±2)/MJ·kg^−1^	41.6	38.0	35.6	33.7	32.5	31.9
PHRR (±0.4)/*t*_ig_/kW·m^−2^·s^−2^	12.1	11.9	9.4	11.7	5.5	6.1
FIGRA (±0.2)/kW·m^−2^·s^−1^	7.1	6.8	5.7	6.0	3.6	3.9
MARHE (±5)/kW·m^−2^	314	301	231	238	154	142

**Table 9:** The phosphorus content of the rubber compounds and in the residue determined by elementary analysis of the residue.

Parameter	EPDM	EPDM/PANI	EPDM/APP	EPDM/APP/PANI	EPDM/APP/ATH	EPDM/APP/ATH/PANI
wt.% P (sample)	-	-	3.24	3.11	2.47	2.39
wt.% P (residue)	-	-	8.46	8.45	6.80	6.21
Phosphorus in the condensed phase/%	-	-	100	100	100	100
Phosphorus in the gas phase/%	-	-	0	0	0	0

**Table 10:** Quantification of the cone calorimeter data of the EPDM rubber composite via calculations of the flame retardancy mode of action: charring, gas phase action, and the protective layer effect.

Material	(1 − μ)	χ·$h_{c}^{0}$	m_0_	Cal. THE	THE	Cal. PHRR	PHRR	Prot. Layer
	%	%	%	%	%	%	%	%
EPDM	100.0	100.0	100.0	100.0	100.0	100.0	100.0	0.0
EPDM/PANI	98.0	91.2	105	93.7	96.8	89.4	88.8	0.7
EPDM/APP	90.0	85.6	110	84.8	83.9	77.0	66.7	13.4
EPDM/APP/PANI	91.0	80.9	112	82.4	81.7	73.7	73.6	0.1
EPDM/APP/ATH	78.0	78.0	127	77.3	69.9	60.9	49.3	19.0
EPDM/APP/ATH/PANI	79.0	76.6	128	77.3	69.9	60.5	49.2	18.8
